# Renal Cyst Rupture Mimicking Grade V Renal Trauma After Blunt Injury: A Case Report Highlighting the Risk of Radiologic Overstaging

**DOI:** 10.7759/cureus.116407

**Published:** 2026-09-17

**Authors:** Victor C Quintana, Cássio Luís L Riccetto, Monique L Dipe

**Affiliations:** 1 Urology, Hospital Centro Médico de Campinas, Campinas, BRA; 2 General Medicine, Independent Physician, Campinas, BRA

**Keywords:** blunt truma, conservative treatment, gross hematuria, polycystic kidney diseases, renal trauma

## Abstract

Blunt renal trauma is commonly assessed using contrast-enhanced computed tomography (CT), which guides management decisions based on injury grading. However, pre-existing renal pathology may confound imaging interpretation and lead to an overestimation of injury severity. We report the case of a previously healthy 26-year-old male patient presenting with flank pain and hematuria after blunt abdominal trauma during a soccer (football) match. Contrast-enhanced CT demonstrated findings suggestive of American Association for the Surgery of Trauma (AAST) grade V renal injury, including apparent parenchymal lacerations, a large perirenal hematoma, and active contrast extravasation. Bilateral renal cysts and hepatic cysts were also identified, raising suspicion for underlying cystic kidney disease. Despite alarming imaging findings, the patient remained hemodynamically stable and was successfully managed conservatively, with no clinical or radiological progression. This case highlights renal cyst rupture as an important differential diagnosis in patients with cystic kidney disease presenting with apparent high-grade renal trauma and emphasizes the importance of integrating imaging findings with clinical stability to avoid unnecessary invasive interventions.

## Introduction

Renal trauma accounts for up to 5% of all trauma cases and represents approximately 24% of abdominal solid organ injuries [1]. The majority of cases result from blunt mechanisms [2]. In this setting, contrast-enhanced computed tomography (CT) is the imaging modality of choice for the diagnosis and staging of renal injuries [1-4], playing a central role in anatomical assessment through the American Association for the Surgery of Trauma (AAST) renal injury grading system [1,5].

Accurate injury classification is particularly important in high-grade renal trauma, as it directly influences management strategies. Higher-grade injuries, especially AAST grade V lesions, are more frequently associated with hemodynamic instability, major concomitant injuries, and higher rates of surgical exploration and nephrectomy [4]. In addition, non-operative management of severe renal injuries should only be performed in highly controlled settings with intensive monitoring, serial clinical and laboratory assessment, and immediate access to diagnostic imaging, interventional radiology, surgery, and blood products [5].

Despite its clinical utility, the AAST grading system is based exclusively on anatomical disruption and does not account for pre-existing renal abnormalities or structural alterations [1,5]. Consequently, underlying conditions such as cystic renal disease may significantly affect imaging interpretation and lead to overestimation of trauma severity, potentially influencing clinical decision-making regarding the feasibility and safety of conservative management [3,6].

In this context, we report a case of renal cyst rupture mimicking AAST grade V renal trauma following blunt abdominal injury, highlighting an important diagnostic pitfall that may impact injury classification and subsequent management decisions.

## Case presentation

A 26-year-old previously healthy male patient presented to the emergency department after sustaining blunt abdominal trauma during a soccer match involving a frontal collision with another player. He reported immediate flank pain but continued playing. Several hours later, he developed worsening abdominal pain and gross hematuria, prompting medical evaluation. He denied prior medical conditions, hospitalizations, medication use, or relevant family history.

On admission, the patient was hemodynamically stable with normal vital signs. Physical examination was unremarkable except for left flank tenderness on deep palpation.

Laboratory investigations, as summarized in Table 1, revealed hemoglobin level of 14.1 g/dL, hematocrit at 41%, leukocyte count at 8,320/mm³, platelet count at 131,000/mm³, serum creatinine at 1.52 mg/dL, and serum urea at 58 mg/dL. Urinalysis demonstrated significant hematuria (>1,000,000 RBCs/mL).

**Table 1:** Laboratory findings during hospitalization NA: not available.

Parameter	Reference Range	Admission	Day 1	Day 2	Day 3	Discharge
Hemoglobin	13-17 g/dL	14.1	13.4	13.4	13.4	13.6
Hematocrit	40-50%	41.0	39.6	38.8	39.1	37.0
Leukocyte count	4,000-10,000/mm³	8,320	5,970	7,150	6,650	5,570
Platelet count	150,000-400,000/mm³	131,000	149,000	146,000	150,000	149,000
C-reactive protein	<10 mg/L	9.1	NA	NA	NA	NA
Prothrombin time	9.4-12.5 s	11.9	NA	NA	NA	NA
Activated partial thromboplastin time	25.1-36.5 s	30.2	NA	NA	NA	NA
Serum urea	10-43 mg/dL	58	30	23	20	18
Serum creatinine	0.59-1.37 mg/dL	1.52	1.10	1.22	1.16	1.14
Red blood cells (RBCs) in urine (urinalysis)	<23,000 RBCs/mL	>1,000,000 RBCs/mL	NA	NA	NA	NA

Contrast-enhanced CT of the abdomen demonstrated normal liver morphology and dimensions, with the presence of hepatic cysts, the largest located in segment VII measuring 14 mm. The right kidney was in normal position, with preserved morphology, contours, and enhancement pattern, showing normal excretion of contrast. Multiple hypodense lesions with fluid attenuation were noted bilaterally, some with thin internal septations, the largest measuring 23 mm in the upper pole of the right kidney and 22 mm in the lower pole of the left kidney. A large left perirenal fluid collection consistent with hematoma was also identified (Figure 1).

**Figure 1:**
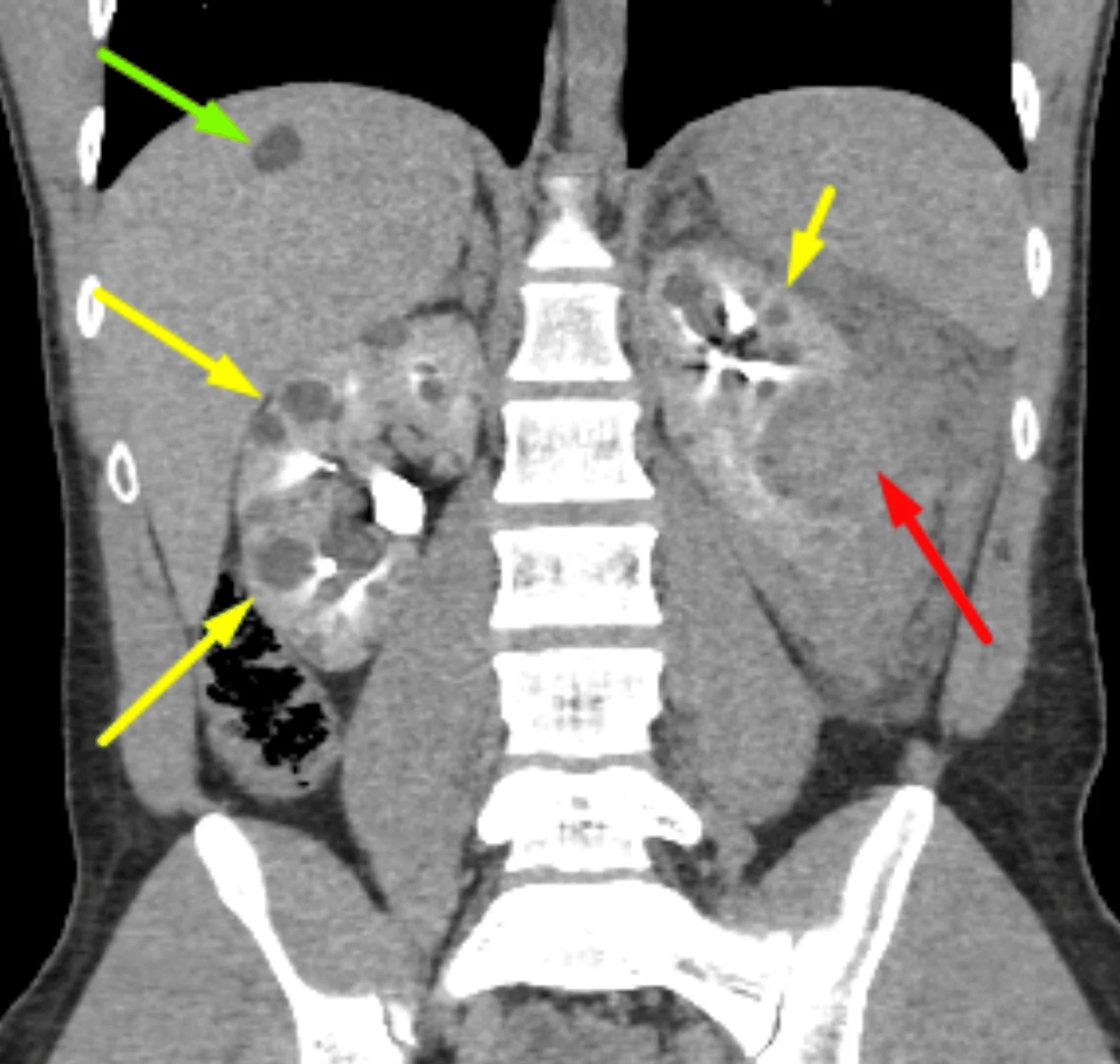
Bilateral renal and hepatic cysts with left perirenal hematoma Coronal contrast-enhanced CT image demonstrating multiple bilateral renal cysts (yellow arrows) and a hepatic cyst (green arrow), consistent with underlying cystic kidney disease. A large left perirenal fluid collection consistent with hematoma is also present (red arrow).

In addition, at least three linear areas of suspected cortical disruption were identified involving the mid and lower poles of the left kidney, associated with a large intrarenal and perirenal hematoma collectively measuring approximately 136×89×84 mm (estimated volume 542 mL). A focus of suspected active contrast extravasation was also observed along the lateral aspect of the lower pole (Figure 2).

**Figure 2:**
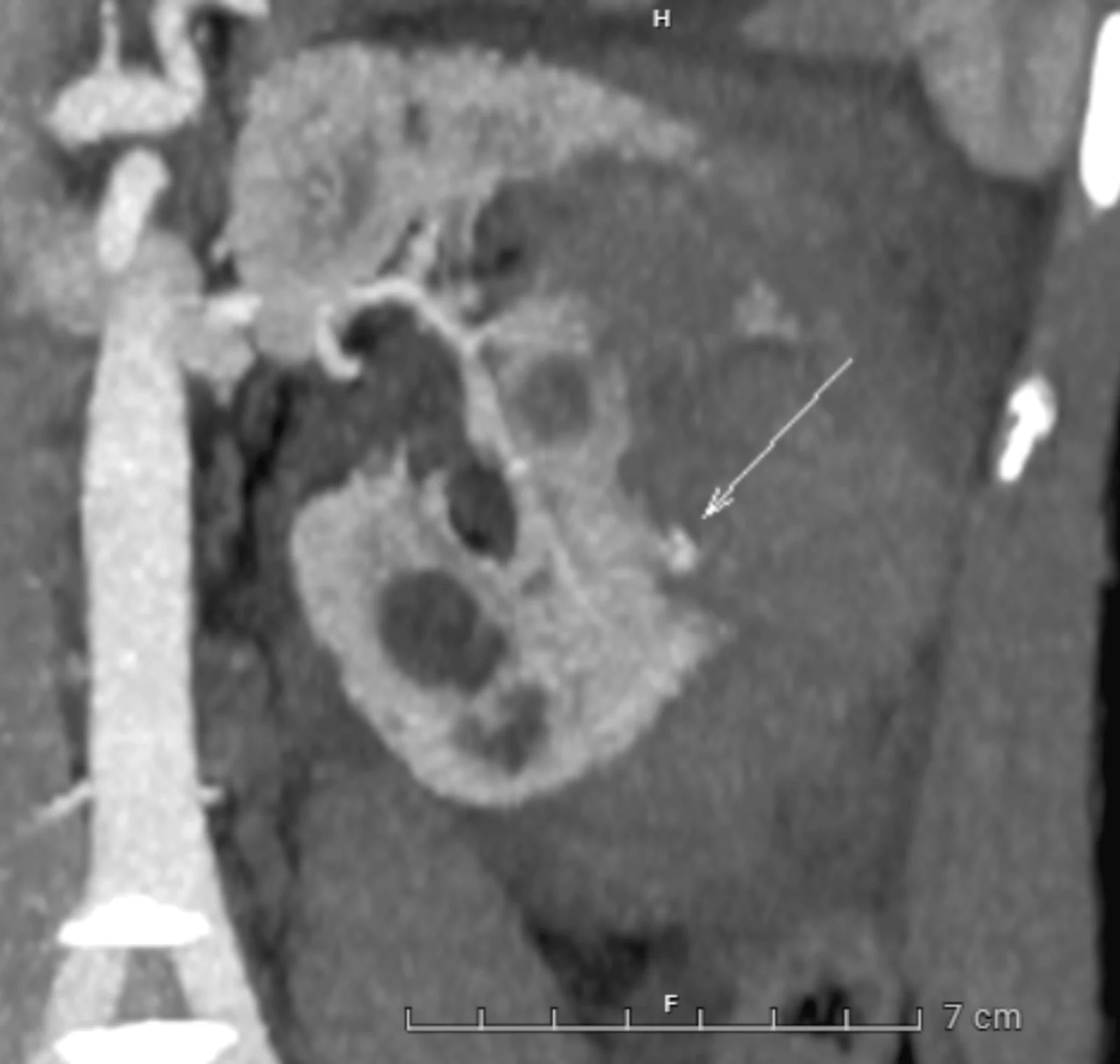
Left renal injury on coronal contrast-enhanced CT Coronal contrast-enhanced CT image demonstrating the left kidney with suspected cortical disruption involving the mid and lower poles. A large left perirenal hematoma is present, associated with a hyperdense fluid collection. The white arrow indicates a suspected focus of contrast extravasation along the lateral aspect of the lower pole.

No clear involvement of the main renal artery or vein was observed, and there were no signs of injury to the collecting system or left ureter.

Radiological findings raised concern for severe renal trauma, particularly AAST grade V injury as initially suggested by radiological interpretation, as well as renal vascular injury, collecting system disruption, and hemorrhage secondary to rupture of pre-existing renal cysts. Despite imaging findings concerning for severe renal trauma, the absence of hemodynamic instability and urinary extravasation suggested a less severe underlying injury.

The patient was admitted to the intensive care unit for close monitoring, and a conservative management strategy was adopted. Management included strict hemodynamic monitoring, serial laboratory testing, and frequent clinical reassessment. Serial physical examinations were performed every four to six hours to evaluate for signs of clinical deterioration, including worsening abdominal pain, peritoneal irritation, or hemodynamic instability.

Supportive treatment consisted exclusively of intravenous tramadol (100 mg every eight hours) for analgesia. No antibiotics, antifibrinolytics, blood products, surgical intervention, or interventional radiology procedures were required during hospitalization. Serial follow-up laboratory findings obtained during hospitalization are also presented in Table 1.

The patient remained hemodynamically stable throughout hospitalization, with no clinically significant decrease in hemoglobin levels (14.1 g/dL on admission vs. 13.6 g/dL at discharge). Repeat imaging after 72 hours demonstrated stability of the hematoma with no progression of bleeding (Figure 3). The patient was discharged after five days of hospitalization and remained asymptomatic at 15-day follow-up.

**Figure 3:**
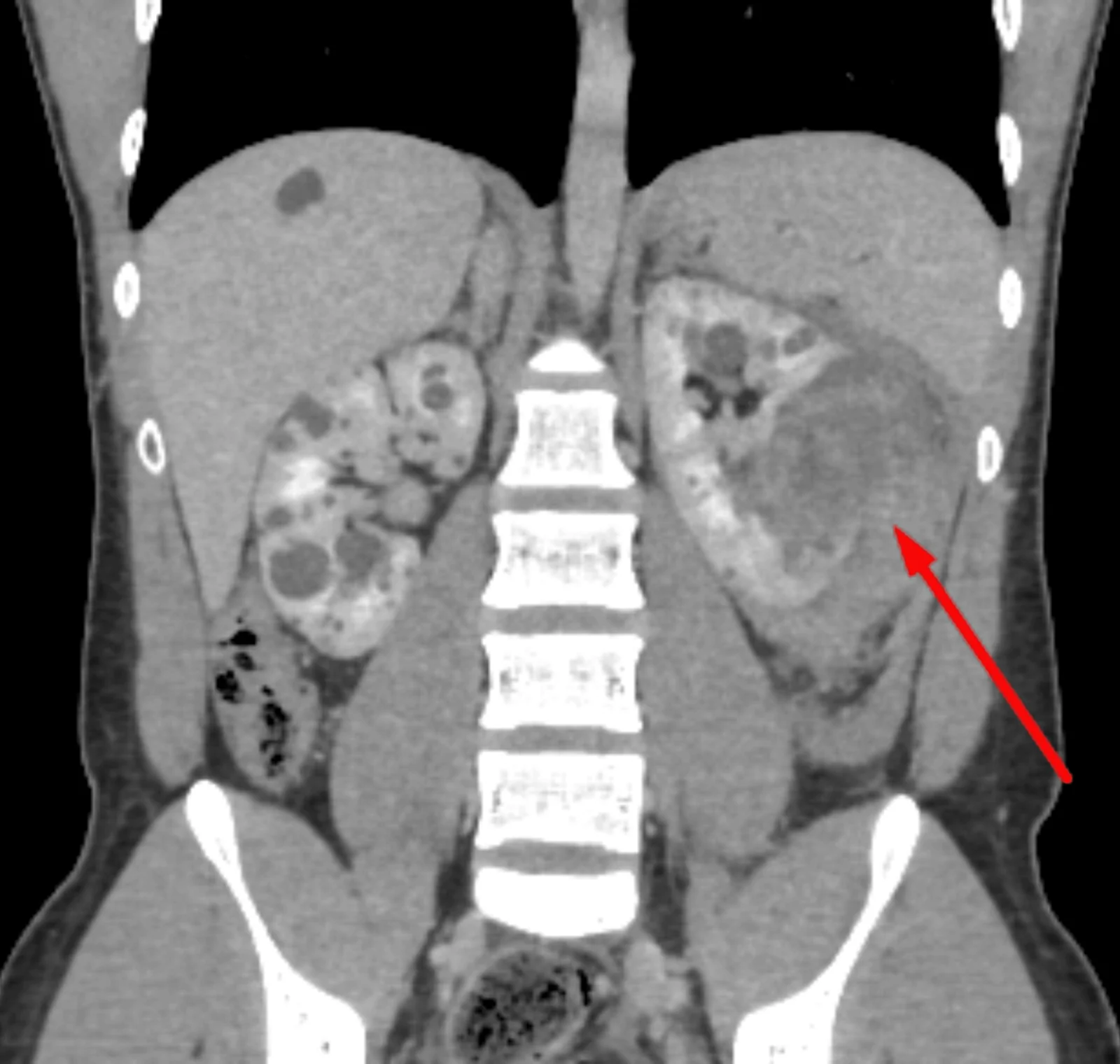
Follow-up contrast-enhanced CT imaging Follow-up contrast-enhanced CT image obtained 72 hours after admission demonstrating stable appearance of the left perirenal fluid collection compatible with hematoma (red arrow), without evidence of active contrast extravasation or progression of bleeding.

## Discussion

This case highlights an important diagnostic pitfall in renal trauma: the potential for underlying cystic kidney disease to result in radiological overstaging on contrast-enhanced CT, mimicking high-grade renal injury. Although CT remains the gold standard for diagnosis and grading of renal trauma, imaging findings must always be interpreted in conjunction with the clinical context [2].

Renal trauma is predominantly caused by blunt mechanisms, and the AAST classification plays a central role in guiding management decisions [1]. However, this system is based primarily on anatomical disruption and does not account for pre-existing renal abnormalities that may lead to diagnostic confusion.

The differential diagnosis in such cases includes true high-grade renal trauma, renal vascular injury, collecting system disruption, and hemorrhage secondary to ruptured renal cysts [3,6]. True grade V injuries typically involve devascularization or major vascular injury, whereas cyst rupture may result in large perirenal hematomas and apparent parenchymal irregularities without significant functional impairment [2,3,5].

Autosomal dominant polycystic kidney disease is the most common inherited renal disorder and is characterized by multiple bilateral renal cysts, frequently associated with hepatic cysts [6]. Cyst rupture is a recognized complication and may present with hematuria, flank pain, and retroperitoneal hemorrhage [3,6].

Importantly, hemorrhage from ruptured cysts may closely mimic severe renal trauma on imaging, including perirenal hematoma and contrast extravasation [2,3]. This overlap may lead to overstaging and potentially unnecessary intervention if clinical findings are not carefully considered.

Despite the imaging appearance suggestive of AAST grade V renal injury, this case illustrates that radiological findings may overestimate injury severity in the presence of underlying cystic kidney disease. In such scenarios, hemodynamic stability, preserved hemoglobin levels, and absence of ongoing bleeding may help differentiate true high-grade trauma from cyst rupture. Careful integration of imaging and clinical findings is essential to avoid misclassification and unnecessary intervention.

## Conclusions

Renal cyst rupture should be considered in the differential diagnosis of patients with cystic kidney disease who present with imaging findings suggestive of high-grade renal trauma. Pre-existing renal abnormalities may lead to radiologic overstaging when trauma classification systems are applied without adequate clinical correlation.

Careful integration of imaging findings with hemodynamic status and overall clinical presentation is essential to avoid unnecessary surgical intervention. In clinically stable patients, conservative management may be a safe and effective approach when close monitoring and appropriate follow-up are ensured.
